# Adenovirus-Vectored Drug-Vaccine Duo as a Rapid-Response Tool for Conferring Seamless Protection against Influenza

**DOI:** 10.1371/journal.pone.0022605

**Published:** 2011-07-27

**Authors:** Jianfeng Zhang, E. Bart Tarbet, Tsungwei Feng, Zhongkai Shi, Kent R. Van Kampen, De-chu C. Tang

**Affiliations:** 1 Vaxin, Inc., Birmingham, Alabama, United States of America; 2 Institute for Antiviral Research, Utah State University, Logan, Utah, United States of America; National Institute of Health, United States of America

## Abstract

Few other diseases exert such a huge toll of suffering as influenza. We report here that intranasal (i.n.) administration of E1/E3-defective (ΔE1E3) adenovirus serotype 5 (Ad5) particles rapidly induced an anti-influenza state as a means of prophylactic therapy which persisted for several weeks in mice. By encoding an influenza virus (IFV) hemagglutinin (HA) HA1 domain, an Ad5-HA1 vector conferred rapid protection as a prophylactic drug followed by elicitation of sustained protective immunity as a vaccine for inducing seamless protection against influenza as a drug-vaccine duo (DVD) in a single package. Since Ad5 particles induce a complex web of host responses, which could arrest influenza by activating a specific arm of innate immunity to impede IFV growth in the airway, it is conceivable that this multi-pronged influenza DVD may escape the fate of drug resistance that impairs the current influenza drugs.

## Introduction

Influenza is a resurging and emerging disease with virtually no possibility of eradicating the causal virus which triggers seasonal as well as pandemic influenza. As a zoonotic disease with the potential to sicken both animals and humans [1], a designer IFV can be rapidly generated by reverse genetics [2] and disseminated by terrorists to ravage agriculture, public health, and economy within a targeted region. Even though this highly contagious and potentially fatal disease has been partially controlled by vaccination, the licensed influenza vaccine is difficult to mass-produce [1] and unable to confer timely as well as broad protection against heterosubtypic IFV strains [3]. Another line of defense against influenza is the use of influenza drugs [*e.g.*, oseltamivir (Tamiflu); zanamivir (Relenza)]; however, this option is limited by the emergence of drug-resistant IFV due to selection under mutational pressure [4], [5].

To develop a rapid-response anti-influenza agent, we serendipitously demonstrated that an Ad5-vectored nasal influenza vaccine could confer rapid protection against influenza in a drug-like manner. A replication-competent adenovirus (RCA)-free Ad5 vector encoding pathogen antigens thus potentially can confer seamless protection against mucosal pathogens as a DVD in a wide variety of clinical settings. RCA-free Ad5 vectors can be rapidly mass-produced in serum-free PER.C6 suspension cells; painlessly mass-administered by nasal spray [1]; followed by elicitation of innate as well as adaptive immune responses in the face of pre-existing Ad5 immunity. In the case of an influenza DVD, the chance to generate drug-resistant IFV is minimal since Ad5 particles conceivably induce an anti-influenza state without directly attacking the IFV. In contrast to a live attenuated IFV vaccine (LAIV), an Ad5-vectored DVD is non-replicating and does not reassort with wild IFV. It is expected that nasal spray of an Ad5-vectored influenza DVD can confer broad protection against heterosubtypic IFV strains for several weeks as a prophylactic drug; followed by elicitation of strain-specific protective immunity as a vaccine for months or even years before the drug-induced protection declines away. This novel regimen may add a rapid-response tool to the public health arsenal against influenza and other diseases if the DVD's protective effects should be reproduced in human subjects.

## Results

### The ΔE1E3 Ad5 particle as an anti-influenza agent

**Figure 1:**
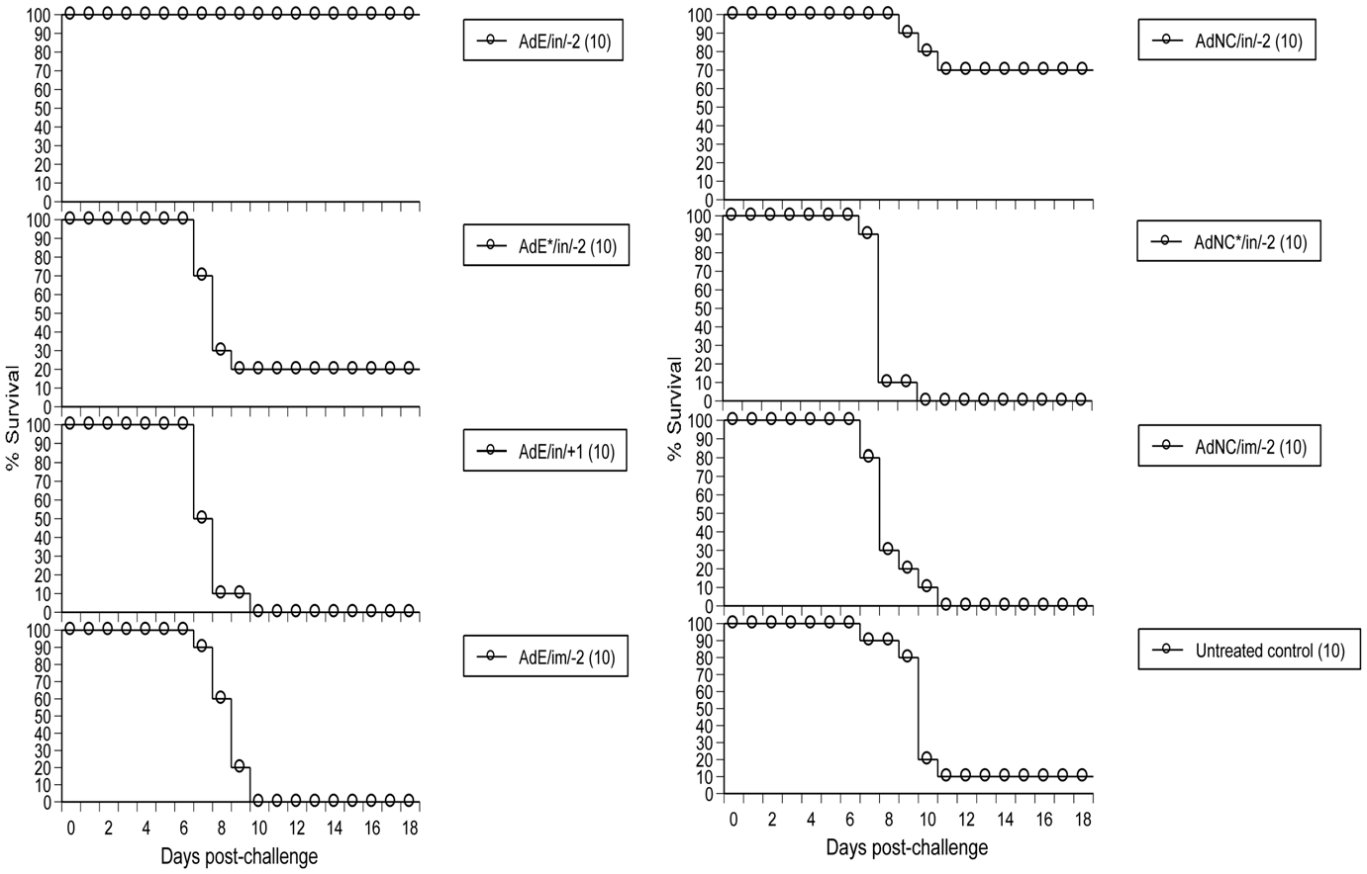
Prophylactic therapy against lethal challenge by PR8 in mice. Prophylactic therapy was performed by i.n. administration of Ad5 particles shortly before PR8 challenge. AdE/in/-2 and AdE*/in/-2, i.n. administration of AdE on day -2; AdE/in/+1, i.n. administration of AdE 1 day post-PR8 challenge; AdE/im/-2, i.m. injection of AdE on day -2; AdNC/in/-2 and AdNC*/in/-2, i.n. administration of AdNC.H1.1 on day -2; AdNC/im/-2, i.m. injection of AdNC.H1.1 on day -2; untreated control, Balb/c mice without treatment prior to PR8 challenge; all groups were inoculated with AdE or AdNC.H1.1 at a dose of 1.7×10^8^ ifu except AdE*/in/-2 and AdNC*/in/-2 groups that received a dose of 1.7×10^6^ ifu; all groups were challenged by i.n. instillation of 4xLD_50_ of PR8 on day 0; body weights were recorded daily for 18 days post-challenge with 30% body weight loss taken as the disease endpoint; numbers in parentheses represent the number of animals in each group.

The transgene-free ΔE1E3 Ad5 empty (AdE) particle and its counterpart AdNC.H1.1 encoding the A/New Caledonia/20/99 H1N1 IFV (NC20) HA1 domain were generated in PER.C6 cells as described [1]. As shown in Fig. 1, i.n. instillation of 1.7×10^8^ infectious units (ifu) of AdE 2 days (day -2) prior to challenge protected 100% (10/10) of mice against a lethal dose of live A/Puerto Rico/8/34 H1N1 IFV (PR8); only 20% (2/10) of the animals were protected when AdE's dose was reduced 100-fold to 1.7×10^6^ ifu; and there was no protection when 1.7×10^8^ ifu of AdE were administered into mice by i.n. instillation 1 day post-PR8 challenge or by i.m. injection on day -2. Insertion of the NC20 HA1 domain into the AdE genome mildly interfered with ΔE1E3 Ad5's capacity to induce an anti-influenza state as only 70% (7/10) of animals were protected when 1.7×10^8^ ifu of AdNC.H1.1 were i.n. administered into mice on day -2. Similar to AdE, neither i.n. instillation of 1.7×10^6^ ifu nor i.m. injection of 1.7×10^8^ ifu of AdNC.H1.1 conferred any protection against PR8 when administered on day -2 (Fig. 1). The protection afforded by i.n. administration of AdE (*P*<0.0001) or AdNC.H1.1 (*P* = 0.0077) at a dose of 1.7×10^8^ ifu on day -2 reached statistical significance when compared to that of the untreated control group (by Log-rank tests).

**Figure 2:**
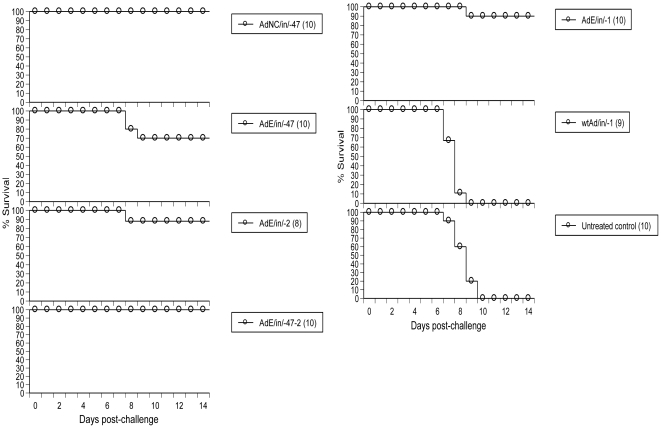
Protection of mice by Ad5-mediated prophylactic therapy and vaccination against a higher dose of PR8 challenge. AdNC/in/-47, i.n. administration of AdNC.H1.1 on day -47; AdE/in/-47, i.n. administration of AdE on day -47; AdE/in/-47-2, i.n. administration of AdE on day -47 followed by a booster application of day -2; AdE/in/-1, i.n. administration of AdE on day -1; wtAd/in/-1, i.n. administration of E1^+^/E3^+^ wild-type Ad5 particles on day -1; all groups were inoculated with Ad5 particles at a dose of 1.2×10^8^ ifu followed by challenge with 10xLD_50_ of PR8 on day 0; body weights were recorded daily for 14 days post-challenge; other symbols and protocols are the same as those described in Fig. 1 legend.

Intranasal administration of AdE on day -47 (47 days prior to PR8 challenge) protected 70% of animals (7/10) showing that the AdE-induced anti-influenza state could persist for several weeks (Fig. 2). Intranasal instillation of AdNC.H1.1 on day -47 protected 100% (10/10) of mice (Fig. 2) presumably due to NC20 HA1-induced adaptive immunity which cross-reacted with PR8 even though no serum hemagglutination-inhibition (HI) antibodies to PR8 were detectable (Table 1). Unlike immunization with AdNC.H1.1 on day -47 which elicited high HI antibody titers to NC20 and undetectable titers to PR8, challenge with PR8 induced high HI antibody titers to PR8 and low titers to NC20 in survivors, and administration of either AdE or AdNC.H1.1 on day -2 induced HI titers to neither NC20 nor PR8 (Table 1). The protection afforded by i.n. administration of AdNC.H1.1 on day -47 (*P*<0.0001), AdE on day -47 (*P* = 0.0032), AdE double-dose regimen (day -47 followed by a booster application on day -2) (*P*<0.0001), AdE on day -1 (*P*<0.0001) or -2 (*P* = 0.0005) at a dose of 1.2×10^8^ ifu all reached statistical significance when compared to that of the untreated control group.

**Table 1:** Serum HI antibody titers induced by AdNC.H1.1 immunization and PR8 challenge.

Immunization	*n*	Day of serum collection	Log_2_[anti-NC20 HI GMT] (±SD)	Seroconversion to NC20 (%)	Log_2_[anti-PR8 HI GMT] (±SD)	Seroconversion to PR8 (%)
aAdNC/in/-2 + PR8	7	19	7.9 (±0.5)	100	8.9 (±0.5)	100
aAdE/in/-2 + PR8	10	19	5.3 (±0.7)	100	7.5 (±0.6)	100
bAdNC/in/-47	10	−1	10.2 (±1.7)	100	2.3 (±0)	0
bAdNC/in/-2	10	−1	2.3 (±0)	0	2.3 (±0)	0
bAdE/in/-2	10	−1	2.3 (±0)	0	2.3 (±0)	0
bUntreated control	10	−1	2.3 (±0)	0	2.3 (±0)	0

HI antibodies were measured against the respective IFV with titers expressed as GMT on a log_2_ scale; a log_2_ titer of 2.3 was arbitrarily assigned to samples with undetectable titers; each serum sample was run in triplicate wells;

a animals described in Fig. 1 with sera collected 19 days post-PR8 challenge;

b animals described in Fig. 2 with sera collected 1 day prior to PR8 challenge. Seroconversion was defined as ≥4-fold rise in HI titer above the preimmune baseline; *n*, number of animals; GMT, geometric mean titer; SD, standard deviation.

**Figure 3:**
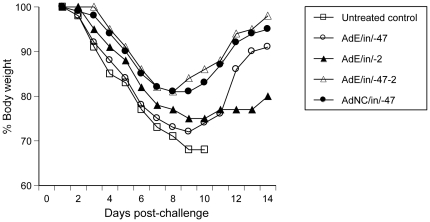
Health status of PR8-challenged animals as shown by body weight loss. Post-challenge body weights are presented as mean % body weight by taking the body weight of individual mice on day 1 as 100%. Symbols and challenge protocols are the same as those described in Figs. 1 and 2 legends. Although AdE/in/-47-2 and AdNC/in/-47 animals lost less weight than mice in other groups, the difference did not reach statistical significance (by one-way ANOVA with Turkey's multiple comparison post-tests; the untreated control group was excluded in statistical analysis due to early termination of data points).

Although several regimens protected mice against influenza-mediated mortality, the AdE double-dose regimen tended to confer more solid protection than its single-dose (day -47 or -2) counterpart as shown by less body weight loss after PR8 challenge even though the difference did not reach statistical significance (Fig. 3). To induce an anti-influenza state, it is essential to delete E1 and/or E3 since the E1^+^/E3^+^ wild-type Ad5 was unable to arrest influenza after i.n. administration into mice under identical conditions (Fig. 2).

### Ad5-induced protection of the lung against influenza

**Figure 4:**
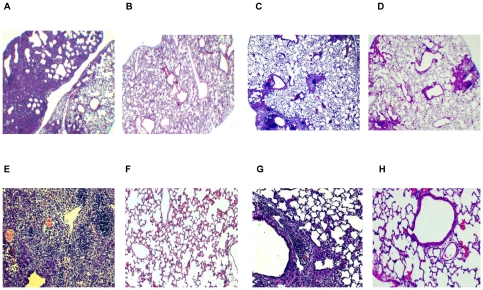
Lung histopathology induced by PR8 infection. (**A** and **E**) Lung resected from an untreated control mouse (Fig. 1) 19 days post-PR8 challenge. (**B** and **F**) Lung resected from a normal Balb/c mouse as a control. (**C** and **G**) Lung resected from an AdE/in/-2 mouse (Fig. 1) 19 days post-PR8 challenge; each section is a representative of three mice. (**D** and **H**) Lung resected from an AdNC/in/-2 mouse (Fig. 1) 19 days post-PR8 challenge; each section is a representative of three mice. Lung sections were examined on a Zeiss Axioskop2 plus microscope using a 2X (**A**–**D**) or a 10X (**E**–**H**) objective lens in conjunction with an Axiocam digital camera.

**Figure 5:**
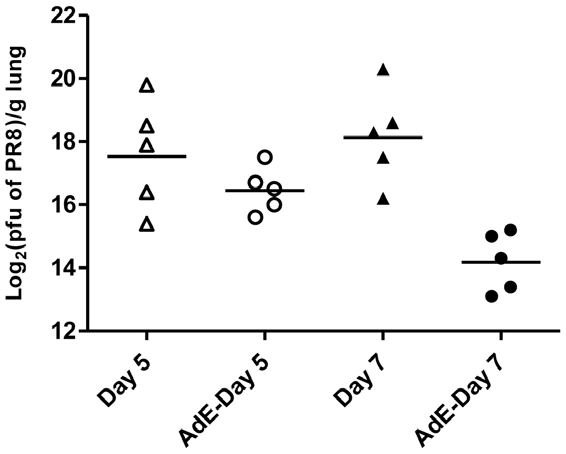
PR8 titers in lungs post-challenge. AdE particles (1.2×10^8^ ifu per 50 µl) were i.n. instilled into mice on day -2 followed by challenging control and AdE-exposed mice with 4.6×10^6^ pfu of PR8 on day 0. Day 5, PR8 titers in lungs resected from control mice 5 days post-PR8 challenge; AdE-Day 5, PR8 titers in lungs resected from AdE-exposed mice 5 days post-PR8 challenge; Day 7, PR8 titers in lungs resected from control mice 7 days post-PR8 challenge; AdE-Day 7, PR8 titers in lungs resected from AdE-exposed mice 7 days post-PR8 challenge; triangle and circle, log_2_(pfu of PR8)/g lung in individual mice; bar, geometric mean of PR8 titers in lungs. No PR8 titers were detected in lungs resected from control mice that were not challenged with PR8. The difference between Day 7 and AdE-Day 7 reached statistical significance (by one-way ANOVA with Turkey's multiple comparison post-tests).

As shown by lung histopathology after PR8 challenge, i.n. administration of AdE or AdNC.H1.1 on day -2 protected mice against influenza by preventing the development of severe lung injuries. Intranasal instillation of PR8 without Ad5 protection induced massive pulmonary inflammation 19 days post-challenge (Fig. 4A) when compared to a normal mouse lung (Fig. 4B). Intranasal administration of AdE (Fig. 4C) or AdNC.H1.1 (Fig. 4D) on day -2 greatly reduced the level of acute lung injury. When the lung sections were examined microscopically under higher magnification, it was visible that PR8 challenge without Ad5 protection induced massive epithelialization of alveolar tissues; multiple foci of monocytes; vascular congestion; early fibrosis; hemorrhage; and perivascular cuffing (Fig. 4E). Prophylactic therapy with AdE or AdNC.H1.1 prevented many of the PR8-induced lung injuries from occurring although perivascular cuffing was still common (Fig. 4G), and healthy blood vessels (Fig. 4H) as well as healthy alveoli (Fig. 4G and H) could be found in the Ad5-protected lungs. Ad5-mediated reduction of lung histopathology was in line with the arrest of PR8 growth in the lungs post-challenge. As shown in Fig. 5, the difference in PR8 titers between the lungs of control and AdE-exposed animals reached statistical significance (by one-way ANOVA with Turkey's multiple comparison post-tests) 7 days post-PR8 challenge.

### Protection against a pandemic IFV strain

**Figure 6:**
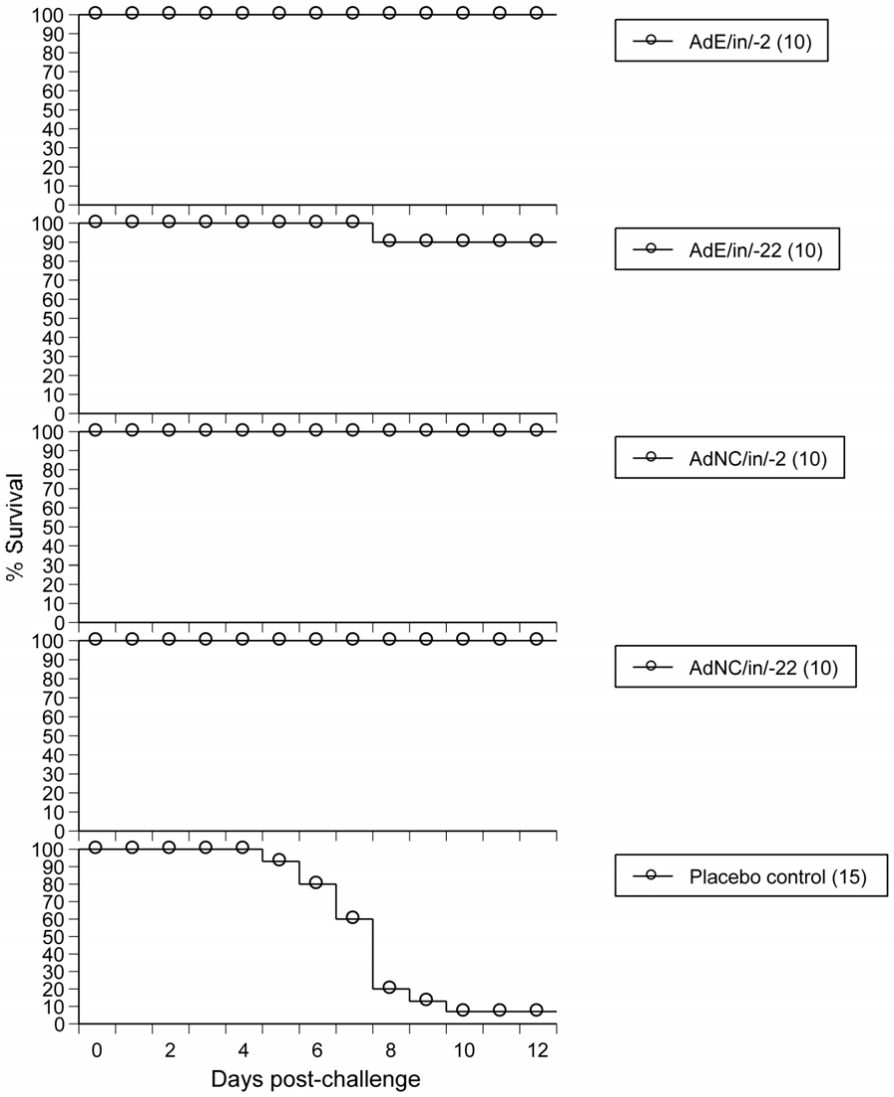
Protection against lethal challenge by the pandemic CA04 in mice. AdE or AdNC.H1.1 particles (2.5×10^8^ ifu per 50 µl) were i.n. instilled into mice at varying time points followed by CA04 challenge. AdE/in/-22, i.n. administration of AdE on day -22; AdNC/in/-22, i.n. administration of AdNC.H1.1 on day -22; placebo control, i.n. administration of 50 µl saline on day -22; animals were challenged by i.n. instillation of 3xLD_50_ of the pandemic CA04 on day 0; other symbols and protocols are the same as those described in Fig. 1 legend.

To demonstrate that ΔE1E3 Ad5 particles can protect mice against not only PR8 but also a more clinically relevant IFV strain, 2.5×10^8^ ifu of AdE or AdNC.H1.1 were i.n. administered into mice followed by challenging animals with a lethal dose of the pandemic 2009 H1N1 swine flu isolate A/California/04/2009 (CA04). As shown in Fig. 6, 100% (10/10) of animals were protected by i.n. instillation of AdE or AdNC.H1.1 on day -2 and AdNC.H1.1 on day -22; 90% (9/10) were protected by i.n. administration of AdE on day -22. The protection afforded against CA04 in all these Ad5-exposed groups reached statistical significance when compared to that of the placebo control group (*P*<0.0001).

## Discussion

The non-replicating ΔE1E3 Ad5 vector has been bioengineered into a nasal influenza vaccine carrier with high potency and excellent safety profile [1]. In addition to the elicitation of protective immunity as a vaccine, we show here that this class of vaccine can also confer prophylactic therapy against influenza before adaptive immunity is elicited. It has been documented that administration of ΔE1E3 Ad5 particles into mice rapidly induces the production of a wide array of inflammatory cytokines and chemokines [6] including type I interferon (IFN-α and IFN-β) [7]; impairs lung dendritic cells [8]; activates natural killer cells [9]; induces production of the antiviral nitric oxide [10]; triggers multi-faceted interactions between Ad5 and blood proteins, platelets, macrophages, endothelial cells, and respective parenchymal cells [6]. Inhibition of Ad5-associated inflammation by Ad5 E1A, E1B, and E3 proteins [11] suggests that the E1^+^/E3^+^ Ad5's incompetence to induce an anti-influenza state (Fig. 2) may be attributed to suppression of inflammation, although other mechanisms cannot be excluded since ΔE1E3 Ad5 particles induce many immune as well as non-immune responses and some reactions remain undefined in animals [12]. It is conceivable that multiple reactions induced by the ΔE1E3 Ad5 particles may integrate for establishing an anti-influenza state in the airway, thus creating a multidimensional defense barrier that can hardly be bypassed by an IFV. This hypothesis is supported by the finding that the IFN-α/β receptor provides protection against influenza in a dispensable manner showing that animals have evolved overlapping mechanisms to respond to influenza [13]. Furthermore, Balb/c mice challenged in these studies carry a defective allele of the IFN-α/β-induced influenza-resistance factor Mx1 [14] implying that the ΔE1E3 Ad5-induced production of type I IFN [7] may not play a major role during the establishment of an anti-influenza state in this mouse strain.

The finding that i.n. administration of AdE 1 day post-PR8 challenge was unable to arrest influenza (Fig. 1) suggests that the IFV may induce a pro-influenza state that is not disrupted by the ΔE1E3 Ad5 particle when the former enters the airway prior to the latter, similar to the Ad5-induced anti-influenza state that cannot be reversed by an IFV when AdE particles were i.n. administered prior to PR8 or CA04 (Figs. 1, 2, 3, 4, 5, 6). To further develop the ΔE1E3 Ad5-based prophylactic drug into a post-exposure influenza drug, it is crucial to characterize the antagonistic reactions induced by the two types of viruses in the airway.

Pre-exposure to Ad5 has been associated with loss of Ad5's potency when this vector is i.m. injected [15]. However, emerging evidence shows that an Ad5-vectored nasal vaccine can bypass pre-existing Ad5 immunity in mice [15], macaques [16], and humans [17] probably due to high-efficiency gene delivery into cells in the superficial layer along the mucosal barrier in conjunction with potent antigen presentation associated with this immunocompetent interface tissue. The synergy between primary and booster applications induced by the AdE double-dose regimen (Figs. 2 and 3) shows that the rapid anti-influenza responses induced by AdE were additive in the presence of pre-existing Ad5 immunity. These findings hold promise that this nasal influenza DVD not only is able to induce rapid and sustained protection against influenza in a single-dose regimen but also may be administered repeatedly (*e.g*., when a different HA is required for its vaccine component) without losing potency.

Although prophylactic influenza therapy can be performed by i.n. administration of complex bacterial lysates [18] or bacterial toxins [19], the bacterial component-induced anti-influenza state was very transient with its protective effects declining within a few days post-therapy [18], [19]. The finding that AdE-induced protective effects could persist for at least 3 weeks (Fig. 6) and up to 47 days (Fig. 2) in a single-dose regimen suggests that the underlying mechanisms between bacterial component- and Ad5-induced anti-influenza states may differ. In addition, only the latter would allow sufficient time for the DVD's vaccine component to elicit adaptive immunity before its drug effects decline away. Moreover, the replicating wild-type Ad5 is a benign respiratory virus and its non-replicating counterpart used in this study should be even safer; notably, the safety profile of an Ad5-vectored nasal influenza vaccine in human subjects has been shown [17]. As a common respiratory virus, the human mucosal immune system is familiar with Ad5 particles and must have evolved Ad5-specific protective mechanisms. In contrast, administration of a digestive tract-associated bacterial toxin into the respiratory tract as an influenza drug [19] would surprise the immune system and this unnatural regimen has been associated with the induction of Bell's palsy in human subjects [20].

The IFV is insidious in mutating into drug-resistant strains when it is attacked by an influenza drug [*e.g.,* the M2 ion channel blocker (amantadine; rimantadine) or the neuraminidase inhibitor (oseltamivir; zanamivir)] [5]. Unlike contemporary influenza drugs, the Ad5-vectored DVD conceivably changes the habitat in the respiratory tract without directly attacking the IFV; hence the DVD confers no mutational pressure to induce drug resistance. In contrast to the oseltamivir-induced suppression of mucosal immunity with the risk to enhance vulnerability to subsequent mucosal pathogen infections [21], the Ad5-vectored DVD enhances mucosal innate immunity against at least a subset of mucosal pathogens. The DVD's efficacy is further fortified by its vaccine component that elicits sustained adaptive immunity before its drug effects completely disappear (Figs. 1, 2, 3, 4, 5, 6). Since the licensed LAIV (*e.g.,* FluMist® in the U.S.) contains live IFV [1], co-administration of LAIV with an influenza drug would be counter-productive because the drug would disable the vaccine by killing live IFV. The Ad5-vectored DVD not only is compatible with a licensed influenza drug, but also it confers prophylactic therapy as a drug by itself in addition to its vaccine capacity.

Emerging evidence shows that a number of nasal vaccines induce a weaker systemic adaptive immune response than their parenteral counterparts [22]-[26] even though nasal vaccines confer more robust protection against a respiratory mucosal pathogen by eliciting a more potent mucosal adaptive immune response [22], [25]. We provide evidence that not only adaptive immunity but also innate immunity could be induced with a focus on the respiratory tract against mucosal pathogens when the ΔE1E3 Ad5 particle is administered i.n. but not i.m., as shown by % survival afforded by i.n. and i.m. routes, respectively (Fig. 1). Whether the Ad5-vectored nasal DVD can confer protection against influenza induced by other routes (*e.g.,* oral infection) remains to be seen.

The finding that i.n. administration of AdNC.H1.1 on day -47 induced more robust protection against PR8 challenge than its counterpart inoculated on day -2 or AdE administered on day -47 (Figs. 1 and 2) suggests that animals in the AdNC/in/-47 group may be protected by an NC20 HA1-mediated adaptive immune response that cross-reacted with PR8 47 days post-immunization in the absence of detectable serum HI antibody to PR8 (Table 1). The data corroborate other reports that serum HI antibody titer is an inadequate surrogate marker for predicting protective immunity induced by a nasal influenza vaccine [24], [26].

The findings that the Ad5-vectored DVD can confer prophylactic therapy in conjunction with vaccination in a single package provide a foundation for the development of a novel anti-influenza agent that can be mass-produced in cultured cells, administered painlessly by nasal spray, with the capacity to bypass pre-existing Ad5 immunity and mobilize the innate as well as the adaptive immune repertoires toward a rapid and sustained beneficial response against influenza, without the potential to generate drug-resistant IFV strains.

## Materials and Methods

### Adenovirus

To generate the AdE particle, homologous recombination between the shuttle pAdHigh and the Ad5 backbone pAdEasy-1 plasmids was performed in *Escherichia coli* BJ5183 cells followed by generation of the RCA-free AdE particle in PER.C6 cells (provided by Crucell Holland BV; Leiden, The Netherlands) as described [1]. AdE is thus a ΔE1E3 Ad5 with an expression cassette in its E1 region [1] without encoding any transgene. To generate the AdNC.H1.1 vector, the NC20 HA gene was synthesized at GENEART (Regensburg, Germany) with codons optimized to match the tRNA pool found in human cells in conjunction with the insertion of a eukaryotic ribosomal binding site immediately upstream from the initiation ATG codon [27]. The NC20 HA1 fragment containing 347 amino acids was amplified from the synthetic HA template by polymerase chain reaction (PCR) using primers 5′-CACAGGTACCGCCACCATGAAGGCCAAGCTG-3′ and 5′-GAGTCTAGATTATCAGCCGAACAGGCCTCTGCTCTGG-3′. The KpnI-XbaI fragment containing the amplified HA1 fragment with a stop codon added in-frame was inserted into the KpnI-XbaI site of pAdHigh in the correct orientation under transcriptional control of the human cytomegalovirus (CMV) early promoter. An RCA-free Ad5 vector encoding the NC20 HA1 (AdNC.H1.1) was subsequently generated in PER.C6 cells as described above. Both AdE and AdNC.H1.1 were validated by DNA sequencing; mass-produced in PER.C6 cells; purified by ultracentrifugation over a cesium chloride gradient as described [27]; dialyzed into A195 buffer [28] with titers (ifu per ml) determined in 293 cells [17] by the Spearman-Karber method [29] after staining Ad5-infected monolayers with a horseradish peroxidase (HRP)-conjugated anti-Ad5 hexon antibody and the 3,3′-diaminobenzidine tetrahydrochloride (DAB) substrate (Clontech Laboratories, Inc.; Mountain View, CA). The E1^+^/E3^+^ wild-type Ad5 (VR-1516) was obtained from the American Type Culture Collection (ATCC; Manassas, VA).

### Influenza virus

PR8 (VR-95) was obtained from the ATCC and grown in Madin Darby Canine Kidney (MDCK) cells in the presence of TPCK-trypsin as described [17] with titers determined by plaque assay [30]. The mouse-adapted CA04 was generated by Natalia A. Ilyushina and provided by Elena Govorkova at the St. Jude Children's Research Hospital (Memphis TN). The CA04 virus was adapted to replication in the lungs of Balb/c mice by 9 sequential passages through mouse lungs. Virus was plaque purified in MDCK cells and a virus stock was prepared by growth in 10-day-old embryonated chicken eggs and then MDCK cells as described [31] with titers expressed as cell culture infectious doses (CCID_50_) as described [32]. NC20 was provided by the Center for Disease Control (CDC; Atlanta, GA).

### Challenge studies

Intranasal administration and i.m. injection of 50 µl of Ad5 particles into young (approximately 2 months old) female Balb/c mice were performed as described [27]. Mice were challenged by i.n. instillation of 50 µl of PR8 containing either 1.4×10^6^ plaque-forming units (pfu) [equivalent to approximately 4xLD_50_ (50% lethal dose)] or 3.5×10^6^ pfu (equivalent to approximately 10xLD_50_) at University of Alabama at Birmingham (UAB), as well as 90 µl of CA04 containing 2×10^5^ CCID_50_ (equivalent to approximately 3xLD_50_) at Utah State University (USU). All experiments using mice were performed in accordance with the approval of the Institutional Animal Care and Use Committees at UAB and USU (UAB Approval ID, #7705; UAB Animal Welfare Assurance Number, A3255-01; USU Approval ID, #552; USU Animal Welfare Assurance Number, A3801-01). Animal facilities at both UAB and USU have been AAALAC accredited.

### PR8 titers in lungs post-challenge

AdE particles were i.n. administered into young female Balb/c mice at a dose of 1.2×10^8^ ifu in a volume of 50 µl on day -2. Five to seven days after i.n. instillation of 4.6×10^6^ pfu of PR8 on day 0, control and AdE-exposed mouse lungs were immediately frozen on dry ice after resection and stored at −80°C until analysis. After thawing, a fraction of each lung was weighed and homogenized in cold phosphate buffered saline (PBS) as a 10% (w/v) suspension. Tissue debris was removed by centrifugation and the supernatant was transferred to another sterile tube for virus titration. Plaque assay of IFV was performed as described [30].

### Hemagglutination-inhibition assay

Sera were tested for activity against PR8 or NC20 by standard HI assay after pre-treatment of the sera with a receptor-destroying enzyme as described [17]. Each serum sample was tested beginning at a dilution of 1∶10. All sera were tested in a blinded fashion on code-labeled, matched pre- and post-immunization samples. Animals were considered seronegative and assigned an HI antibody titer of 5 (2.3 on a log_2_ scale) if their serum specimen had an HI titer of <10.

### Lung histopathology assay

Mouse lungs were fixed by perfusing 10% buffered formalin through the trachea. Paraffin-embedded tissues were cut into 5- µm-thick slices followed by staining sections with hematoxylin and eosin.

### Statistical analysis

All statistical analysis was performed using GraphPad Prism version 5.04 (GraphPad Software, San Diego, CA). Log-rank tests were performed for comparing Kaplan-Meier survival curves; and one-way ANOVA with Turkey's multiple comparison post-tests were performed for comparing body weight loss as well as PR8 titers in lungs. Statistical significance was set at *P*<0.05.
